# Designing siRNAs against non-structural genes of all serotypes of Dengue virus using RNAi technology – A computational investigation

**DOI:** 10.1016/j.jgeb.2025.100523

**Published:** 2025-06-23

**Authors:** Md. Nur Islam, Israt Jahan Asha, Aninda Kumar Gain, Raihanul Islam, Shipan Das Gupta, Md. Murad Hossain, Shuvo Chandra Das, Mohammed Mafizul Islam, Dhirendra Nath Barman

**Affiliations:** a Department of Biotechnology and Genetic Engineering, Noakhali Science and Technology University, Noakhali-3814, Noakhali, Bangladesh; b Centre for Learning in Advanced Bioinformatics (cLAB), Noakhali- 3814, Noakhali, Chattogram, Bangladesh

**Keywords:** siRNA, Dengue virus, Gene silencing, RNAi technology, Molecular therapeutics

## Abstract

Dengue is a viral disease caused by *Aedes aegypti* and *Aedes albopictus* mosquitoes, leads to severe health complications, including dengue hemorrhagic fever (DHF) and dengue shock syndrome (DSS). The disease has intensified in Asian countries such as Bangladesh, India, Myanmar, and Thailand, with regular outbreaks since 2015, partly due to the lack of effective vaccines or treatments against this ferocious virus. Nonstructural (NS) genes of Dengue virus (DENV) are believed to play vital role in viral replication. Targeting the NS genes of existing serotypes of DENV, we aimed to design a potential small interfering RNA (siRNA) that has the capability to silence the NS genes hence, provide a ground strategy for antiviral therapeutics. In this investigation, a comprehensive computational approach encompassing data collection from NCBI database, GC content analysis, conservation prediction across all DENV serotypes, mRNA-siRNA duplex thermodynamic assessment, siRNA efficacy evaluation, molecular modeling and structural refinement, molecular docking were performed. These analyses anticipated three efficient siRNAs (S2, S3 and S11) that could have the capability to silence all the NS protein-coding genes employed by the DENV. Based on additional analysis including molecular dynamic (MD) simulation, principal component analysis (PCA) and free energy landscape (FEL), two siRNAs (S2 and S11) were recommended as potential therapeutics that could effectively degrade viral NS protein-coding genes through the RNAi pathway. However, S2 (Guide: 5′-UGUUUUUCGCCUUUUUCCGUU-3′ and Passenger: 5′- CGGAAAAAGGCGAAAAACACG-3′) molecule is supposed to be appeared the most promising siRNA compared to S11. This study is the foundational contribution enabling chemically synthesized future antiviral drug discovery for combating DENV, especially in the Asian region where frequent outbreaks are common. Nonetheless, *in vivo* validation and further evaluations are necessary before these siRNAs can be advanced as molecular therapeutics.

## Highlights

• Identifying two highly possible siRNAs to silent all non-structural protein coding genes of Dengue virus.
• One siRNA was inspected as an efficient and putative antiviral drug to silence all non-structural genes.
• This siRNA could possibly immunize all serotypes of Dengue viruses. Hence, a future antiviral therapeutic is anticipated.

## 1 Background

The dengue virus (DENV) is a flavivirus that causes dengue, a systemic viral infection, posing a severe threat to human health. This mosquito-borne viral infection is one of the dominant public health concerns in tropical and subtropical regions.^1^, ^2^ The primary vector is the female *Aedes* mosquito, while infected individuals serve as secondary hosts. DENV is primarily spread by day-biting *Aedes aegypti* and *Aedes albopictus* mosquitoes, leading to clinical manifestations ranging from classical dengue fever (DF) to more severe forms such as dengue hemorrhagic fever (DHF) and dengue shock syndrome (DSS). These viruses require an intermediate carrier who carries previously infected blood. Later, the carrier may capable to transmit to any other healthy person via dengue replication. DENV carries a single-stranded positive RNA of around 11 kb in size with methylation on the 5′ ends and polyadenylation on the 3′ ends. This single stranded RNA can translate directly into protein without requiring any transcriptional event, enabling a silent replication by encoding three structural proteins (nucleocapsid C, membrane-associated M, and envelope protein E). However, seven types of non-structural proteins (NS1, NS2A, NS2B, NS3, NS4A, NS4B, and NS5) have significant contribution in the course of infection.^3^, ^4^ Non-structural proteins not only play an important role in viral replication but also in the effect of multifunctional host responses. For example, NS1 jointly works with NS4A/B to enhance the event of viral replication. NS3 directly involves in helicase activity and to cut at certain regions in order to synthesize viral proteins. *NS5* gene is the dominant and the most conserved region in all serotypes that activate RNA-dependent RNA polymerase (RdRp) and RNA methyltransferase (MTase) activity during polyprotein translation.^5^, ^6^

The dengue epidemic was first observed in 1943, mainly in Asia, Africa, and the Americas, and was first reported in Bangladesh in 1964.^7^, ^8^ There are four serotypes of dengue virus (DEN-1 to DEN-4) that are capable of causing fatal conditions in humans. The complications may vary in severity from acute self-limiting febrile illness to life-threatening DHF and DSS. Among them, DENV-2 is the most fatal compared to the others, while DENV-3 was reported main culprit during an outbreak from 2000 to 2024 in Bangladesh.^9^, ^10^ Currently, more than 128 countries annually suffer from the DENV which is becoming a major source of illness and causing life-threatening complications to approximately half of the world’s population.^11^ Thus, the geographic range of dengue is expanding and becoming a major public health issue in many countries like Bangladesh. Moreover, due to the lack of effective strategies, scientists all over the world hardly find specific remedies to manage the recurrence of diseases like dengue or COVID-19.^12^ More concerning, there are no effective antiviral drugs or any universal remedies that can potentially inhibit the viral infections. Since some antiviral drugs are available in the world but not approved for using in many other countries, including Bangladesh. In addition, several studies have shown the specificity of the antiviral agent is one of the narrowing scopes to manage dengue patients.^13^, ^14^

In this context, we aimed to predict and construct putative siRNA to effectively immunize seven non-structural proteins. siRNA molecules have been shown several advantages as antiviral therapeutics. For instance, Amber M. Paul et al., (2014) showed a distinct outcome by inhibiting dengue proliferation *in vitro* through siRNA nanoparticle applications.^15^, ^16^, ^17^ Considering all the amazing promises of siRNA, a comprehensive computational investigation was implemented to design a potential siRNA as therapeutic against dengue proliferation in this current study.

In siRNA-based therapeutics, the RNA interference (RNAi) mechanism relies on key proteins such as Dicer, the RNA-induced silencing complex (RISC), and Argonaute-2 (AGO-2). Generally, Dicer initiates the RNAi pathway, which is mainly responsible for cleaving the long double-stranded RNA (dsRNA) molecule into a short dsRNA fragment (∼23nt) called siRNA. This siRNA allows unwinding the sense and antisense strands in the presence of ATP. The antisense strand of siRNA plays an important role as a guide RNA and is able to bind with the non-structural protein-coding mRNA strands with the help of RISC. This episode induces cleavage of viral mRNA with the help of the Piwi domain present in the AGO-2 protein. This protein found in human genome is a key component of RISC and is responsible for cleavage activity.^18^ Thus, all proteins including Dicer, RISC, and AGO-2 has a significant contribution in the RNAi mechanism which may enable a natural cellular process to inhibit the translation of viral mRNA.

Considering such a significant biochemical strategy, many studies have been reported on targeting certain antigen from a specific serotype and/or strain of the dengue virus for preventing dengue.^19^, ^20^, ^21^ Unlike the conventional study, we considered all the serotypes and strains available in the NCBI database for designing the most effective siRNAs. Therefore, these siRNAs are supposed to immunize all non-structural protein targets. In addition, molecular docking against human AGO-2 protein, molecular dynamic simulation and other relevant study revealed very significant insights to reach a precise conclusion. We believe that the current findings will be helpful to develop antiviral therapeutics against the dengue virus differing from traditional drugs. Therefore, it will open up the opportunity to combat all serotypes of dengue after subsequent *in vitro* experimental validation.

## 2 Materials and methods

**Fig. 1:**
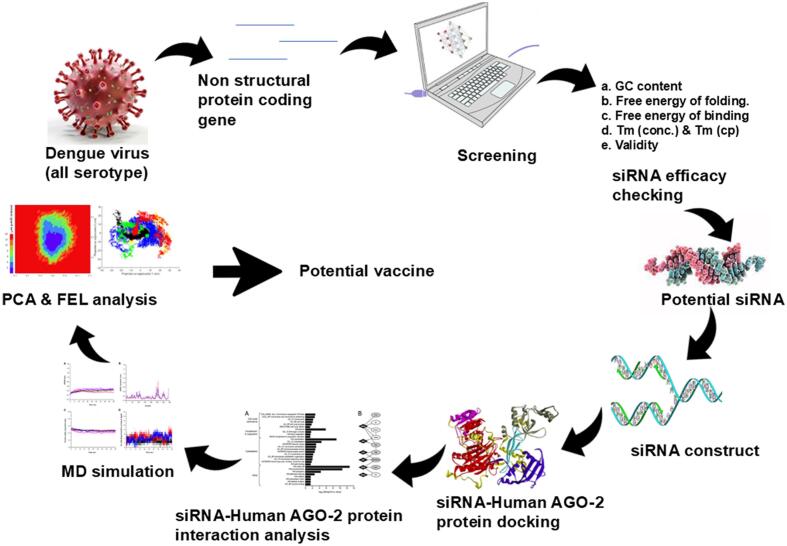
Graphical abstract that conceptualizing the whole experiment.

A complete overview of the methodology for predicting and constructing siRNA-dependent therapeutics against non-structural protein-coding genes of dengue virus is illustrated in the graphical abstract as illustrated in Fig. 1.

### 2.1 Selection and retrieval of DENV genome sequences

A total of 29 complete set of genome sequences of the dengue viruses were retrieved from the NCBI nucleotide database (https://www.ncbi.nlm.nih.gov/nuccore) using the keyword ‘dengue virus-complete genome’. The selected genome sequences averaged ∼ 10173 base pairs (bp) in length. During selection process, we mainly targeted the coding sequence (CDS) along with its conservation. Selected genome shared the common CDS, including *NS1, NS2A, NS2B, NS3, NS4A, NS4B,* and *NS5* which were observed between 2400 to 10450 bp. Genome sequences were carefully downloaded in FASTA format and deposited for further procedures.

### 2.2 Evolutionary study and off-target effect exploration

A comprehensive phylogenetic study was carried out for analyzing the evolutionary relationship among 29 strains of all serotypes of DENV. Preliminary, the whole genome was used to predict the sub-lineage conservancy using MEGA 11 software. In this case, the neighbor-joining method was employed to construct a phylogenetic tree followed by the Tamura-Nei’s algorithm using a 1000 bootstrap value.^22^ Besides, T-Coffee tool (version 11.0) was used to predict the sequence similarity among the selected siRNA molecules containing 23 nucleotides based on their homology score.^23^ This conservancy assessment clarified the homology pattern of the targeted sequences. The highly homologous sequences were subjected for off-target identification. To accomplish this, a nucleotide BLAST (BLASTn) (https://blast.ncbi.nlm.nih.gov/Blast.cgi) was carried out against a human genomic and transcript database. This approach anticipated the exact complementary sequences within the source which could be an unexpected off-target. These off-targets in human genome were considered and excluded on the basis of E-value.

### 2.3 Prediction of target sequences and selection of siRNAs

Prediction of target sequence is essential for designing the most effective siRNA. To achieve this, we employed the siDirect2.1 web server (https://sidirect2.rnai.jp) to predict the possible targets and their respective siRNAs. This server performs various algorithms including Ui-Tui, Reynolds, and Amarzguioui and combinations of all rules were followed during target selection as summarized in Table 1.^24^, ^25^, ^26^ To activate seed-dependent off-target inhibition, a lower seed duplex melting temperature (T_m_) is strictly maintained as the lower T_m_ reduces the chance of off-target effects that helps to avoid non-specific binding.^27^ In this context**,** the T_m_ was set below 21.5 °C which is a default value. The T_m_ was calculated by the following equation:

(2.3) $$T_{m}\;=\;{\left(1000\times \Delta H\right)/\left(A+\Delta S+Rln\left(\frac{\mathrm{CT}}{4}\right)\right)-273.15+16.6log[Na}^{+}]$$

**Table 1:** Guidelines followed for designing siRNA molecules.

Serial no.	siRNA design guidelines proposed by	Guidelines
1.	Amarzguioui et al. (2004)	1. Reference for the 5′-end of the siRNA duplex, where there is a differential binding favoring A or U at the 3′ end of the antisense strand. 2. position 1 of the sense strand (the strand that corresponds to the target mRNA sequence) should not contain a U 3. The sense strand should have strong binding with the antisense strand at the 5′ end to ensure that the siRNA duplex is stable and correctly loaded into the RISC. 4. In case of 3′ sense strand will regarded as weak binding.
2.	Reynolds et al. (2004)	1. GC content range of 30–52 % ensures balanced thermodynamic properties for efficient binding to the target mRNA (1 point) 2. Occurrence of Three or More A or U Base Pairs at Positions 15–19 of the Sense Strand (1 point) 3. Little Internal Stability at Target Site (Tm > 20 °C) (1 point) 4. Occupancy of U at Position 10 of the Sense Strand (1 point) 5. Occupancy of A at Position 3 of the Sense Strand (1 point) 6. Occupancy of A at Position 19 of the Sense Strand (1 point) 7. Absence of G at Position 13 of the Sense Strand (1 point) 8. Threshold for efficient siRNAs: Score ≥ 6
3.	Ui-Tei et al. (2004)	1. 5′ end of the sense strand should start with either (A) or (U) 2. 5′ end of the antisense strand should begin with either (G) or (C) 3. The first 7 nucleotides of the sense strand in 5′ terminal should have a minimum of 4 (A) or (U) 4. Should avoid having a continuous stretch of GC pairs that exceeds 9 base pairs in length.

As seen in the equation (2.3)^,^

• ΔH represents the total amount of change of nearest neighbor enthalpy in kcal/mol
• A is the helix initiation constant (−10.8)
• The sum of the nearest neighbor change is represented by ΔS
• R is characterized by the gas constant (1.987 cal/deg/mol)
• CT is the total molecular concentration (100 µM) of the strand and
• [Na^+^] indicates the concentration of sodium, which is fixed at 100 mM

In addition, Guide strands of siRNA identified using siDirect and i-score designer web tools were cross-verified by using s-Biopredsi, and DSIR algorithms, which play second-generation prediction techniques.^28^, ^29^, ^30^, ^31^ This identification provides a ground validity to select the target sequences recommended by several datasets.

### 2.4 Assessment of parameters for siRNA-guide strands refinement

Several refinement procedures were implemented to find out the most effective siRNAs relevant to eleven target sequences reported by siDirect and i-score designer. At the beginning of the refinement, Guanine-Cytosine (GC) content of siRNA molecules in percentage was calculated through the Oligocalc web server.^32^ The secondary structure and folding free energy of siRNA molecules were predicted using the MaxExpect tool from the RNAstructure web server, which identifies most probable nucleotide pairings for structural accuracy.^33^ Different folding energy produces on the basis of different structure. Optimum level of folding energy and accurately formulated secondary structures were considered for further evaluation.

### 2.5 Establishment of the siRNA-mRNA complex based on thermodynamics

The thermodynamics assessments of siRNA-mRNA complexes were critically examined, as it plays a crucial role in RNAi mechanism. Optimal thermodynamics indicated strong binding affinity where lower free energy is expected. To assess this, siRNAs were hybridized with their target mRNAs using the Bifold tool in the RNAstructure suite, which calculates the free energy of intramolecular base pairing during complex formation.^33^ Meanwhile, the heat capacity of melting temperatures T_m_ (C_p_) and concentration-melting temperatures T_m_ (Conc.) were measured by using the DINA Melt webserver (https://www.unafold.org/hybrid2.php). In this case, the 5′→3′ direction of the guide strand of selected siRNAs was used for thermodynamics calculation. The melting temperature T_m_ is the form of a function of temperature that expresses the components of the ensemble of the heat capacity (C_p_). Furthermore, T_m_ (Conc.), the melting-concentration temperature may be computed to detect the point at which the concentration of the siRNA-mRNA complex starts to half its initial maximum value. A defined concentration plot was examined for cross-validation to determine the specific T_m_ (C_p_).

### 2.6 Computation of efficacy for designed siRNAs

Effectiveness testing is mandatory for successful silencing of the target mRNA sequences. To accomplish this, two macroscaled databases, siRNAPred (https://webs.iiitd.edu.in/raghava/sirnapred/index.html) and siPRED, were employed followed by the Hybrid-7 estimation method.^34^ The siRNAPred database predicts experimentally valided siRNAs based on a dataset comprising 2,182 siRNAs across 21 experimental studies, utilizing a support vector machine (SVM) algorithm for prediction. Moreover, this dataset ranked the list of siRNAs with three predicting scores, e.g., “very high efficacy”, “high efficacy”, and “moderate efficacy”, followed by a range of above 1, 0.8–0.9, and 0.7––0.8, respectively. Similarly, the siPRED dataset produces a siRNA inhibition efficacy score where a higher percentage is anticipated for good prediction. Finally, both datasets were cross-validated by the selected siRNAs to get the precise and accurate results.

### 2.7 Molecular docking of selected siRNAs and AGO-2 protein

The purpose of the molecular docking of selected siRNA with the human Argonaute protein is to estimate the holding capacity during the interference gene silencing mediated approach. There are four types of AGO-2 proteins found in the human genome, ranging from AGO-1 to AGO-4. Particularly, AGO-2 is responsible for specific gene silencing at the post-transcriptional or transcriptional level by the formation of loaded siRNA complexes.^35^ The AGO-2 protein was retrieved from the Protein Data Bank (PDB ID: 4Z4D) and prepared for docking using PyMOL software.^36^ Later, energy minimization was calculated through the YASARA energy minimization server. The most putative siRNAs were subjected to constructing their 3D structures through RNAcomposer- a server used for RNA construction through complete automation (https://rnacomposer.cs.put.poznan.pl). In this case, an interactive mode for a single 3D-RNA structure model was used along with the RNAstructure prediction method. After generating 3D models of siRNA, we minimized their structural geometry using UCSF Chimera.^37^ 1000 steepest descent steps along with 100 conjugate gradient steps and FF99SB from the AMBER force field were applied to refine and minimize the structures. We employed the HDOCK server to perform docking, in which every docking complex was built with its binding energy.^38^ Docking complexes along with their binding poses were visualized using PyMOL, UCSF chimera. Structural summary obtained from the docking complexes were analyzed using the PDBsum server (https://www.ebi.ac.uk/thornton-srv/databases/pdbsum/).

### 2.8 Analysis of molecular dynamics (MD) simulation

MD simulation is the method in which structural compatibility and stability of protein–ligand complexes are assessed through specific time steps. We simulated the human AGO-2 and siRNA complexes to uncover the insightful information about their compactness and relevant dynamic behaviors. In this case, GROMACS version 2024.4 with a specific system (Gigabyte Technology B560M; Processor: Intel® Core^TM^ i7-10700 CPU @ 2.90 GHz having 16 cores; GPU: NVIDIA Geforce GTX 1650; RAM: 2 × 16 GB DDR4; Disk capacity: 1 × 1.3 TB SSD; OS name: Ubuntu 24.04 LTS) was employed.^39^ The AMBER99SB-ILDN force field along with the TIP3P water models was parameterized for this action. Proper solvation and neutralizations were achieved by deploying sufficient Na^+^ and Cl^−^ ions. Moreover, additional Mg^2+^ ions were used to stabilize the phosphate backbone into nucleic acid. In the energy minimization step, the steepest descent minimization algorithm was executed, followed by 50,000 steps having a maximum force of a 10 kJ/mole. Furthermore, NVT (constant Number of particles, Volume and Temperature) and NPT (constant Number of particles, Pressure and Temperature) equilibration were ensembled at 200 ps and captured the trajectories with a 2 fs time steps. 300 K of temperature and 1.0 bar of pressure were maintained during NVT and NPT equilibrations, respectively. Finally, production MD of 150 ns was executed with a 2 fs time steps while structural coordination was observed and captured the trajectories after finishing every 10 ps. The significant trajectories were then analyzed to predict and evaluate structural compactness and conformational stability.

### 2.9 Principal component analysis and computation of free energy landscape

Principal component analysis (PCA) was used to identify major collective motions of the AGO-2-siRNA complex during the 150 ns simulation. This technique explores insights into conformational changes and binding mechanisms by reducing the complexity of the data. Moreover, this statistical method is also used to uncover the overall energy profile of the system using free energy landscape (FEL).^40^ Therefore, we calculated both PCA and FEL to find the dynamic behavior and overall stability of the AGO-2-siRNAs complexes during simulation. In this case, we utilized g_covar and g_anaeig protocols available in the GROMACS package. All the significant trajectories were tuned to eliminate rotations and translations to assess the covariance matrix. After diagonalizing the matrix, we created a 2D projection of eigenvalues and plotted through two PCs (PC1 and PC2). The principal eigenvectors for capturing the most significant motions were computed and plotted onto the multidimensional space, followed by Cartesian trajectory coordinates. Moreover, the g_sham module of GROMACS computed the Gibbs free energy landscape to assess the thermodynamic stability.

## 3 Results

### 3.1 Sequence retrieval and conservancy analysis

**Fig. 2:**
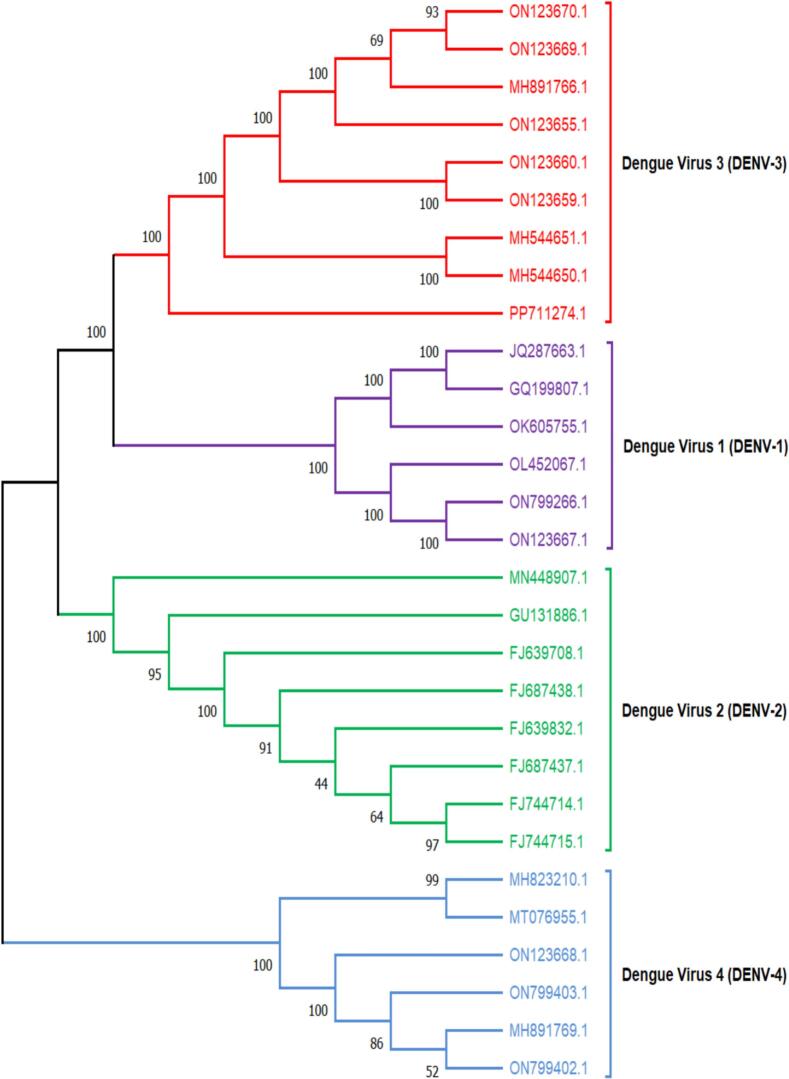
Phylogenetic tree construction for analyzing conservancy pattern among all the serotypes of DENV using 1000 bootstrap value. DENV: Dengue virus.

A total of 29 complete genome sequences representing available serotypes of dengue virus (DENV-1 to DENV-4) were retrieved from NCBI, with data curated on 22 November 2024. After downstream analysis, 11 strains were identified that share the non-structural protein-coding genes in their genome, known as *NS1, NS2A, NS2B, NS3, NS4A, NS4B, and NS5*. These 11 strains were subsequently used for siRNA prediction, aiming to design therapeutics molecules capable to immunize all of them. Meanwhile, a phylogenetic tree using 29 strains was constructed to assess the genetic divergences among them. In this analysis, a remarkable divergence was observed, with DENV-4 identified as an out-group. Overall, the strains exhibited relatively low divergence, supported by bootstrap values exceeding 70 % as illustrated in Fig. 2.

### 3.2 Selection of siRNA target through various parameters

Initially, 61 siRNA molecules were shortlisted based on the various parameters from both the siDirect and thei-score designer datasets. These datasets apply various algorithms, allowing them to be more object-oriented. Notably, in the i-score designer, no candidate was considered with i-score, i-Biopredsi, and DSIR scores below 0.8. Accordingly, a series of screenings of all the aforementioned sequences was conducted. After considering several parameters, ultimately 11 target sequences were identified as the most promising candidates through a cross-validation approach as shown in Table 2. To avoid off-target effects, we considered higher number of E-value obtained from BLASTn ranging from 0.03 to 199 in both genomic and transcripts datasets. However, higher number of E-value which indicates a lesser or/and weaker evolutionary homology among the targets (human genome and transcripts) and siRNA molecules since they maintained dissimilarity in sequences. Therefore, these siRNA molecules might be avoided unnecessary bindings (off-target) to the intended targets during injecting them into human body for therapeutic purposes, hence might be safer for therapeutic use.

**Table 2:** Sirnas and target mrna of ns protein coding genes anticipated from ncbi.

**siRNA**	**siRNA target into mRNA**	**Target position within mRNA**	**GC content (%)**	**Energy score of folding (kcal/mole)**	**Free energy of duplex (kcal/mole)**	**Thermodynamics (˚C)**		**Efficacy score**
						**T_m_(C_p_)**	**T_m_ (Conc.)**	
S1	GTGGTTTATGATGCAAAATTTGA	7287	29	2.6	−34.3	80.9	79.4	0.837
S2	AACGGAAAAAGGCGAAAAACACG	87	42.1	1.8	−36.7	86.9	85.7	0.874
S3	TTGGATTTCGAACTGATAAAAAC	1037	31.6	1.8	−34.4	83	84.5	0.909
S4	TGGATTTCGAACTGATAAAAACG	1038	29	1.8	−34.0	82.6	81.2	0.847
S5	CACCAATATATGGCTAAAATTGA	2893	29	−3.6	−34.9	80.8	81.5	0.814
S6	GGGACAATTGAAGAAGAATAAGG	307	33	1.8	−37.3	81.7	83.1	94.14
S7	TAGCTTTTTCGCTCATAAAGAAT	9269	33	−3.0	−37.0	83.1	84.1	92.22
S8	AGGCAATTTGGTCCAAATTGAGA	1318	38	−3.1	−39.6	84.9	86.1	0.874
S9	GGGCTAATTTTGCTAAAAATAGT	3866	30	1.7	−34.4	79.9	81.1	0.791
S10	TAGCTTTAATTGCAACATTTAAA	3796	29	−0.3	–32.7	78.1	79.6	0.915
S11	AGGCAATCAAGAGACGTTTAAGA	5248	36.8	1.9	−39.3	85.8	87.2	0.918

### 3.3 Computation of GC content and structural refinement of siRNA candidates

**Fig. 3:**
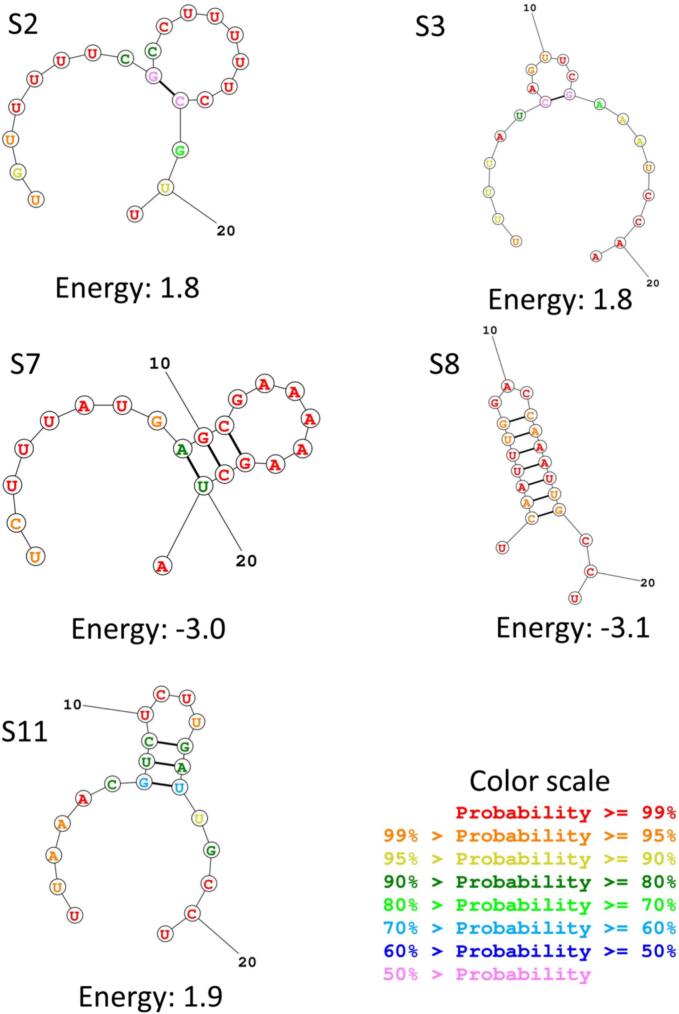
Free energy calculation for siRNA guide strands. Color scale indicates the probability of the nucleotide position coordination with other nucleotides. siRNA: small interfering RNA.

Among the screened 11 siRNAs, three siRNA molecules (S1, S4 and S9) were identified with lower GC content (29–30 %) than the recommended threshold, therefore not considered for further study. Because, less than 30 % as well as higher than 65 % of GC content may interfere with siRNA efficacy. Actually, optimum level of GC content requires for efficient performance of the RNAi mechanism.^41^ Furthermore, four siRNAs (S5, S7, S8 and S10) were excluded as they demonstrated negative folding free energy as displayed in Fig. 3 and Table 2. The negative free energy of folding indicates the siRNA molecule requires higher energy making it inefficient in terms of optimum binding.^42^ The remaining four siRNA (S2, S3, S6, and S11) molecules demonstrated high potential for further assessment.

### 3.4 Efficacy verification followed by their thermodynamics

**Fig. 4:**
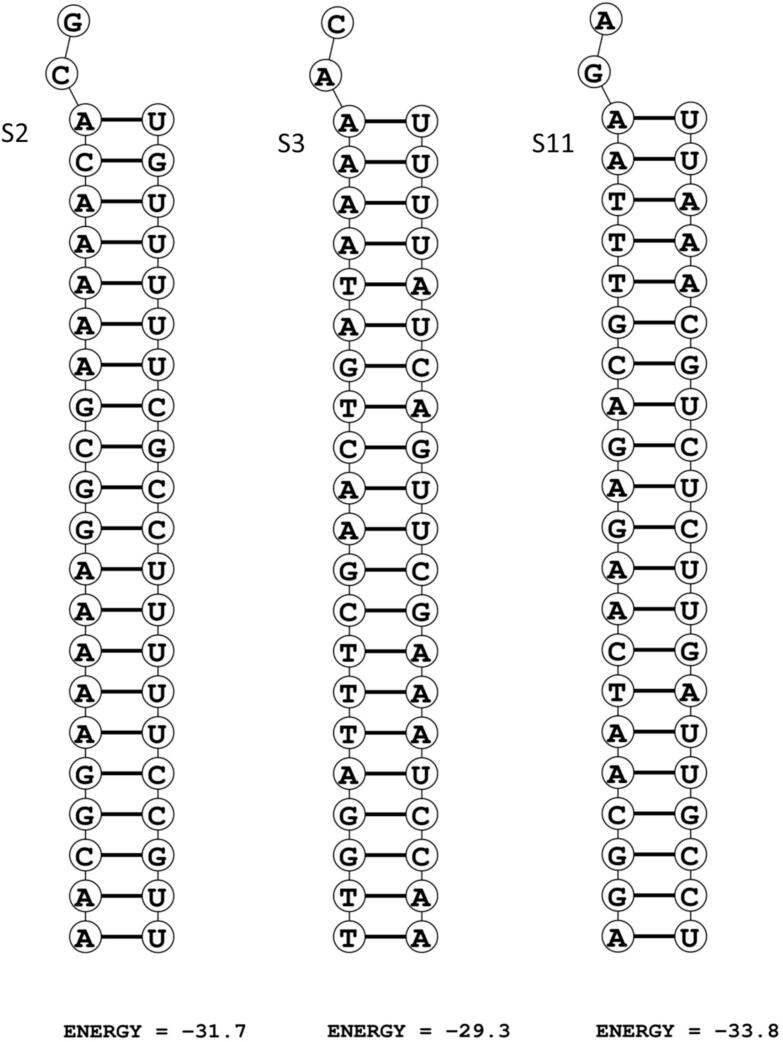
Duplex formation between target mRNA and guide strands of siRNA for calculation of free energy of bonding. Two nucleotides are over hanged while forming a successful duplex. siRNA: small interfering RNA.

The intracellular efficacy of RNAi is the most vital factor, as it determines the ability of the selected siRNA to effectively silence its complementary target sequences. This event depends on various cofactors including thermodynamics and efficacy status. Considering these facts, S2, S3, and S11 were selected based on their lowest energies in duplex formation between the guide strand of siRNA and the target mRNA. The calculated binding free energies for the corresponding duplexes were −34.3, −36.7, and −34.9 KJ/mol for S2, S3, and S11, respectively, as illustrated in Fig. 4. Furthermore, heat capacity of the melting temperature plot, T_m_ (C_p_), and concentration-melting temperature plot, T_m_ (conc.), after formation of siRNA-mRNA complex were computed more than 85˚C in both cases, which satisfies the optimum level. In addition, the siRNApred and the siPRED were employed to verify the predicted efficacy of the selected siRNAs by comparing with experimentally validated siRNAs. S2, S3 and S11 showed more than and 85 % of inhibition efficacy and higher than 0.85 of efficacy score is considered to be high as mentioned in Table 3. However, the various indexes of S6 molecule in terms of T_m_ (C_p_), T_m_ (conc.) and efficacy scores were observed as 81.7˚C, 83.1˚C and 0.84, respectively which indicate the overall stability was not promising. Therefore, S6 molecule was not considered for further study.

**Table 3:** Computation of essential parameters of the siRNA candidates against Dengue virus.

**SiRNA**	**siRNA target position in conserved sequence**	**Target position within mRNA**	**siRNA sequences** **(Guide)** **(Passenger)**	**siRNAprd predicted Efficacy Score** **(Hybrid-7)**	**siPRED predicted Inhibition Efficacy** **(%)**	**Seed duplex instability (T_m_) (˚C)**		**Free energy of duplex** **(kcal/mole)**	**GC content (%)**
						**Guide**	**Passenger**		
S2	85–107	87	UGUUUUUCGCCUUUUUCCGUU CGGAAAAAGGCGAAAAACACG	0.874	87.74	8.7	14.3	−34.3	42.1
S3	1035–1057	1037	UUUUAUCAGUUCGAAAUCCAA GGAUUUCGAACUGAUAAAAAC	0.909	94.47	8.9	16.7	−36.7	31.6
S6	305–327	307	UUAUUCUUCUUCAAUUGUCCC GACAAUUGAAGAAGAAUAAGG	0.84	94.14	6.2	12.1	−37.3	33
S9	3864–3886	3866	UAUUUUUAGCAAAAUUAGCCC GCAAUUUGGUCCAAAUUGAGA	0.874	94.71	−9.7	−4.3	−34.4	30
S11	5246–5268	5248	UUAAACGUCUCUUGAUUGCCU GCAAUCAAGAGACGUUUAAGA	0.918	93.5	15.0	13.8	−34.9	36.8

### 3.5 Molecular modeling and docking analysis

**Fig. 5:**
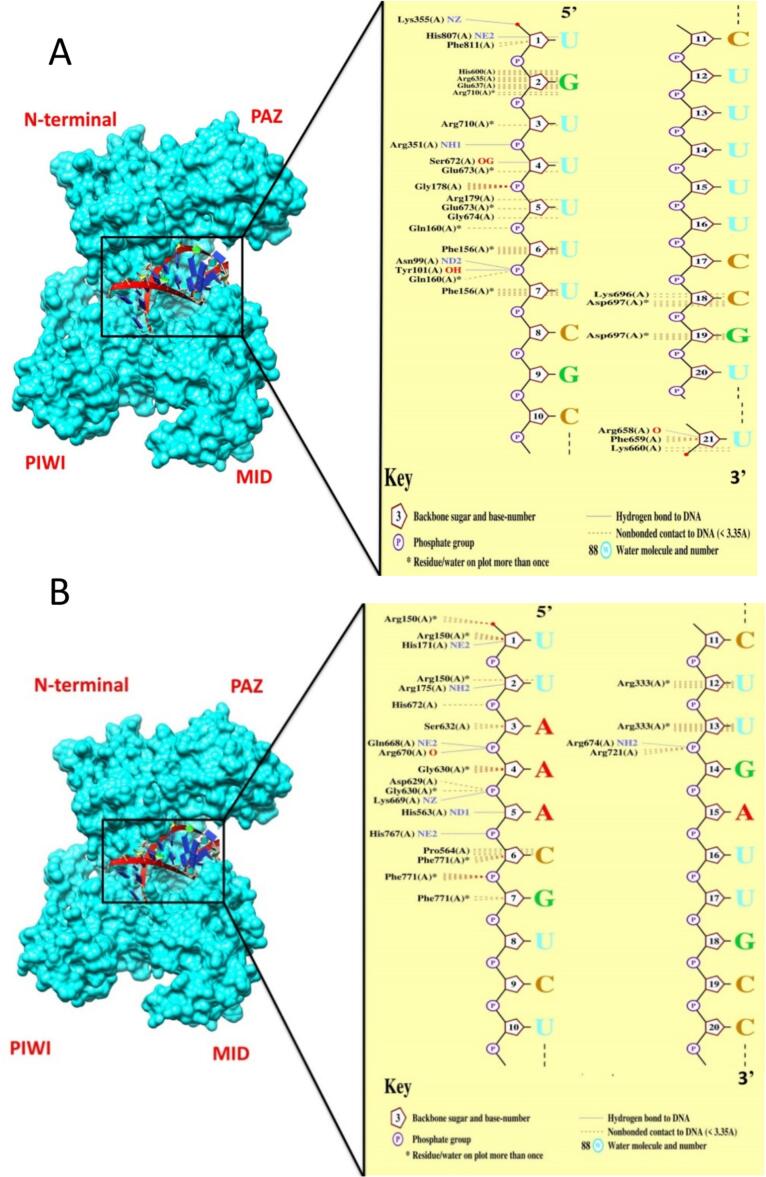
Docking analysis between human AGO-2 protein and selected siRNA molecules. (A) Represents the interacting residues of AGO-2 and siRNA-2 complex. (B) Represents the interacting residues of AGO-2 and siRNA-11 complex. AGO-2: Argonaut- 2.

After a series of screening, the three siRNA molecules (S2, S3 and S11) are remaining and subjected for docking with human AGO-2 protein. In this step, their 3D structures were generated from an RNA composer tool. After docking, all the necessary docking statistics were calculated, including docking score, motive prediction of the receptor, and interacting residues as shown in Fig. 5. S2 and S11 molecules showed −365.17 and −319.78 of docking score, respectively. In contrast, S3 molecule showed less favorable docking score of −207.54, indicating weaker binding affinity compared to other molecules (S2 and S11), hence is not considered for MD simulation. The interacting residues for human AGO-2 protein, including GLY^178^, ARG^179^_,_ LYS^355^, HIS^807^, PHE^811^, HIS^600^, ARG^710^, ARG^351^, SER^672^, GLY^674^_,_ and HIS^563^ are matched with previous studies.^43^, ^44^, ^45^ Therefore, these residues are supposed to be conserved and showed common in binding with siRNA molecules as shown in Table 4.

**Table 4:** Interacting residues observed from molecular docking between siRNA and human AGO-2 protein.

Alias	siRNA sequences (5′- Guide strand- 3′ 5′- Passenger strand- 3′)	Docking score	siRNA − AGO-2 interacting residues in domains			
			N-terminal (64–131)	L1 region (141–188)	PAZ (200–338)	PIWI (349–771)
S2	UGUUUUUCGCCUUUUUCCGUU CGGAAAAAGGCGAAAAACACG	−365.17	ASN^99^ TYR^101^	GLY^178^#ARG^179^# GLN^160^ PHE^156^	N/A	LYS^355^# HIS^807^# PHE^811^**#** HIS^600^# ARG^635^#GLN^637^ ARG^710^# ARG^351^# SER^672^# GLU^673^ GLY^674^# LYS^696^ ASP^697^ ARG^658^ PHE^659^ LYS^660^
S11	UUAAACGUCUCUUGAUUGCU GCAAUCAAGAGACGUUUAAGA	−319.78	N/A	ARG^150^ HIS^171^ ARG^175^	ARG^333^	HIS^672^ SER^632^ GLN^668^ ARG^670^ GLY^630^ ASP^629^ LYS^669^ HIS^563^# HIS^767^ PRO^564^ PHE^771^ ARG^674^ ARG^721^

^#^Represents amino acid residues observed in the previous reports.^43^, ^44^, ^45^

### 3.6 Trajectory analysis from molecular dynamics (MD) simulation

**Fig. 6:**
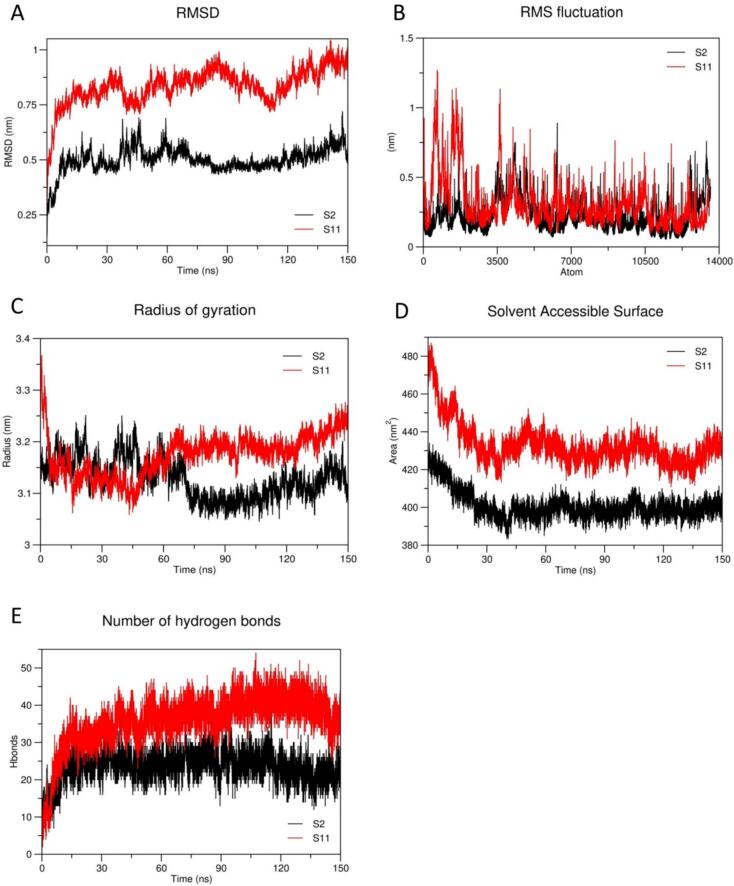
Molecular dynamic (MD) simulation for 150 Ns of both siRNA-2 and siRNA- 11 with human AGO-2 protein act as a receptor. (A) Root Mean Square Deviation (RMSD) plots. (B) Root Mean Square Fluctuation (RMSF) plots. (C) Radius of Gyration (Rg) plots. (D) Solvent Accessible Surface Area (SASA) plots. (E) Number of Hydrogen Bonds (Hbonds) plots. AGO-2: Argonaut- 2, Ns: Nanosecond.

Molecular dynamics simulation is a technique to understand the dynamic behavior of the complexes through computational assessments. Different parameters, e.g. Root Mean Square Deviation (RMSD), Root Mean Square Fluctuation (RMSF), Radius of Gyration (Rg), Solvent Accessible Surface Area (SASA), and Number of Hydrogen bonds (Hbond) were considered. In the RMSD calculation, a comparative analysis was examined between the S2 and S11 complexes, revealing distinct pattern of carbon-alpha atom fluctuations. S11-AGO2 complex showed the RMS deviation value having 1.02 nm, which is higher than that of S2-AGO2 complex, having 0.56 nm. As shown in the Fig. 6A, the S2 complex stabilized after 60 ns, maintaining a consistent fluctuation pattern until the end of the simulation. In the RMSF analysis, the S11 complex showed a higher fluctuation level than the S2 complex. Specifically, the atoms up to position 3500 in the S11 complex displayed greater flexibility, with more pronounced fluctuations. In contrast, the S2 complex exhibited increased flexibility between atoms 3500 and 3800, though fluctuations remained below 1 nm as shown in Fig. 6B. Furthermore, the radius of gyration (Rg) was also measured to understand the compactness of complexes, revealing clear differences in structural stability and folding behavior. Initially, up to 64.58 ns, both the S2 and S11 complexes showed slightly similar patterns of Rg value; however, after 65 ns, the direction of both the complexes started too dissimilar. This observation was helpful to find S2 as more compacted than the S11 complex as illustrated in Fig. 6C. In addition, SASA was also performed to evaluate the accessibility of the solvent of both complexes. In this case, again S2 complex exhibited a lesser accessible area (average of 400 nm^2^) whereas the S11 complex had demonstrated higher accessible area (average of 427 nm^2^). Interestingly, the findings from the SASA analysis align with the Rg trajectory, showing that S2 form more stable and compact complex as depicted in Fig. 6D. Fig. 6E illustrates, the number of hydrogen atom formation, where S2 generates a lower average number of hydrogen bonds (25) compared to S11, which forms a higher average (36). Based on the overall dynamic behaviors, including the stability and compactness, the S2 (siRNA 2) was selected as more stable candidate compared to S11 (siRNA 11).

### 3.7 Principal component analysis and free energy landscape

**Fig. 7:**
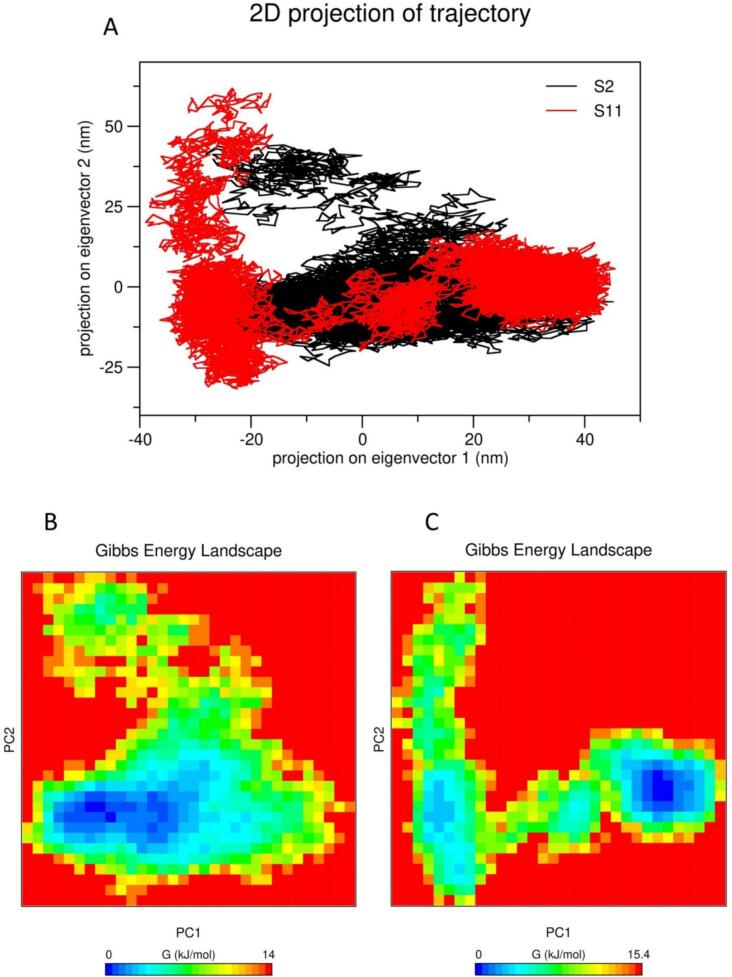
Principal component analysis (PCA) and free energy landscape (FEL) analysis of both complexes of siRNA-2 and siRNA-11 and human AGO-2 protein. (A) 2D projection of the most significant eigenvectors distribution through each direction of PC1 and PC2. (B) Gibbs free energy landscape of siRNA-2 and AGO-2 complex. (C) Gibbs free landscape of siRNA-11 and AGO-2 complex. PC1: principal component 1, PC2: principal component 2, AGO-2: Argonaut- 2.

Principal components of the dynamics and associated free energy were analyzed to understand the total contribution of the molecular motions in each direction. Specifically, two eigenvectors (PC1 and PC2) were plotted through two-dimensional spaces to evaluate the dynamic behavior of the two siRNA-AGO2 complexes. Following matrix diagonalizing, the largest motion distributions were calculated as 1,742.58 nm^2^ for S11 and 851.29 nm^2^ for S2, as shown in Fig. 7A. At the same time, Gibbs free energy landscape (FEL) was calculated to understand its thermodynamic behavior during the simulation. The estimated free energy values were 14.0 KJ/mol for S2 and 15.4 KJ/mol for S11. These results provide insight into thermodynamic potentials generated during the dynamic motions in a particular fashion of direction as illustrated in Fig. 7B and 7C.

## 4 Discussion

The historical significance of the dengue infection in South Asia is noticeable from the very beginning of 1964. In 2000, there was a massive dengue outbreak occurred in Bangladesh, resulting in 79 fatalities.^46^ Since then, recurrence of the dengue outbreak has become a routine phenomenon, with a consistently high mortality rate each year. In 2019, a maximum of 179 deaths were recorded, hence, listing the largest outbreak.^47^ Multiple factors, predominantly, rapid urbanization, global warming, prolonged rainy season, could be responsible for such a pattern of outbreak.^48^ Moreover, the prevalence of four serotypes of dengue (DENV-1 to DENV-4) has been observed in Bangladesh, where DENV-2 and DENV-3 have the predominant nature of infection. The incidence of death tolls due to DHF is increasing every year, which is alarming as well as a major concern for the quality of life of the population. Furthermore, limited resources of remedy in both clinical and post-clinical stages make the current situation more lethal and hazardous. Besides, no antiviral or drug to treat dengue fever in the global market except Balapiravir is available. This is a clinically trialed antiviral drug that is believed to target only the NS5 protein, which has not yet been approved by FDA as it causes several side effects.^49^ Therefore, this lacking of effective antivirals against dengue infection initiates the crying necessity to develop worthwhile antivirals to combat dengue infection.

A number of studies have investigated the siRNA-associated gene silencing approach to develop the effective therapeutics against dengue infection. siRNA, however, helps in RNAi-dependent gene silencing mechanisms and becomes a promising tool for next-generation medicine. Moreover, the application of siRNA in the development of drugs and therapeutics has shown an optimistic value by introducing siRNA nanoparticles against several diseases including viral infection, cancer, and genetic disorders.^50^, ^51^, ^52^ Considering the potential of this technique, in this study a comprehensive computational investigation was implemented to anticipate potential siRNAs that could be an opportunistic resource for developing effective broad-spectrum antivirals to combat infection of all serotypes of DENV.

This study targeted all non-structural proteins across all DENV serotypes due to their involvement in the virus’s development and propagation during infection. During the screening process of potential siRNAs, all available algorithms from the siDirect server and i-score designer were considered. Target sequences were identified based on the parameters including GC content, number of nucleotides and seed-duplex instability (T_m_). Highly relevant targets were selected for further evaluation as shown in Table 3. For effective target prediction, the sequence similarity assessed by T-coffee server displayed favorable manner of all parameters, indicating a significant pattern of similarity among relative strains. The higher the T-Coffee Score (TCS), the better the homology pattern.^23^ In our analysis, TCS score was computed 531 indicating the predicted sequences shared homologous conservation among 29 strains.

Moreover, the phylogenetic result of serotypes showed a high bootstrap value (>70 %) signified consistency with the result of sequence similarity analyzed by T-coffee server. Hence, these conserved sequences could be a good target for designing potential siRNA molecules.

To get an effective binding of siRNA with target mRNA and subsequent degradation, it is essential to have the secondary structure of siRNA maintaining an internal loop.^53^ Therefore, secondary structure formation and its refinement were conducted based on free energy of duplex and energy score of folding. These measurements helped to extract data for downstream analyses as shown in Table 2. Seven siRNAs (S1-S4, S6, S9 and S11) out of eleven (S1-S11) showed promising results based on the aforementioned parameters. The remaining four (S5, S7, S8 and S10) molecules showed negative energy of folding, hence were excluded.

GC content is a major property of potential siRNA, with the recommended range of 30 % to 64 %. In this study, seed duplex instability (T_m_) was critically examined with an emphasis on maintaining lower values to enhance silencing efficiency. However, two siRNAs (S1 and S4) were identified with lower GC content (29 %) than the recommended threshold. Besides, S1 and S4 molecules were observed thermodynamically less stable which proportionally affected the efficacy score as shown in Table 2. Consequently, these candidates were excluded from further consideration. Considering these factors, finally five siRNAs (S2, S3, S6, S9 and S11) were selected for further assessments since promising characteristics are reserved as shown in Table 3.

The biochemical thermodynamics of five selected siRNAs was analyzed based on T_m_ (C_p_), T_m_ (conc.) and GC content. Since GC content significantly affects the thermodynamic stability of siRNA molecules and also required for successive silencing, the relationship between GC content and thermodynamic stability must be determined.^54^ Moreover, efficacy verification was assessed by the siPred server which showed S2, S3 and S11 exhibited > 85 % of inhibition and therefore considered as the most effective siRNA choice. The desired level of inhibition is 80 %.^34^ Although, S6 and S9 molecules showed considerable inhibition efficacy, however, other parameters like GC profile, free energy of folding, free energy of duplex and thermodynamics were not promising. Therefore, S2, S3 and S11 were rationally chosen for docking with AGO-2 protein and for further analysis.

Docking analysis was performed to understand how is the bindings taken place between siRNA molecule and human AGO-2 protein. Consequently, S2, S3 and S11 exhibited a docking score of −365.17, −207.54 and −319.78, respectively. Although S2 and S11 experienced promising docking score, however, S3 produced insignificantly higher score compared to S2 and S11. Besides, it is an important consideration that GC content of S3 molecule was observed the lowest (31.6 %) compared to other siRNA molecules (S2 and S11) as listed in Table 3. Given this lower GC content, which may impact thermodynamic stability and silencing efficacy, S3 was not selected for further analysis. Moreover, docking result confirmed that the interacting residues of S2 and S11 corresponded to highly conserved amino acids at the optimal binding site as listed in Table 4. Some of the residues including GLY^178^, ARG^179^, LYS^355^, HIS^807^, PHE^811^**,** HIS^600^, ARG^635^, ARG^710^, ARG^351^, SER^672^, GLY^674^, and HIS^563^ were identified common in other findings.^43^, ^44^, ^45^ Perhaps these interacting residues identified in Linker 1 region (L-1), PAZ domain and Piwi domain of human AGO-2 protein and may commonly participate with siRNA molecules.

Following rigorous screening, S2 and S11 were subjected to molecular dynamic simulation, and different parameters including RMSD, RMSF, Rg, SASA, and Hbonds, were computed over 150 ns. All the parameters indicated that the molecular dynamic stability of S2 is more feasible than the S11 as illustrated in Fig. 6. This finding is consistent with PCA and FEL analysis, where S2 is indicated to be thermodynamically more stable than the S11 complex as shown in Fig. 7. In addition, T_m_ (C_p_) of S2 was observed higher (86.9˚C) than that of S11 (85.8˚C) molecule during the initial thermodynamics assessments. This finding further supports the result from molecular dynamic simulation, where S2 complex exhibited dynamically more stability than that of S11 complex. These observations suggest that T_m_ (C_p_) may proportionally affect dynamic behaviors of the molecule and thus significantly induce overall efficiency of the complexes.^55^ Therefore, considering these key findings, the S2 complex emerges as the most promising and effective siRNA-associated therapeutic candidate. As the field of research is expanded, researchers are trying to find out the proper way to administer the therapeutics in order to confirm the systematic execution of drug discovery. Several vaccine delivery systems have been reported, where Polyethyleneimine (PEI) is the most prominent in the case of siRNA delivery systems.^56^ This drug delivery system may be applicable due to its several advantages. Our designed siRNA molecule having lower molecular weight can be delivered easily using this delivery system and is expected to remain protected from enzymatic and non-enzymatic degradation which require further validation *in vivo*. Furthermore, the PEI method can boost siRNAs to the particular location without losing their potential where viral propagation is encountered.^57^, ^58^ In addition, there is another opportunity to deliver siRNAs into nano-vesicles which is also a powerful tool for successful administration. However, lyophilization may protect the part of nano-vesicles for enhancing the strength of siRNAs effectively.^59^ Blood-Brain-Barrier (BBB) and microvascular endothelial barrier functions are commonly disrupted by dengue infection, resulting in rapid plasma leakage that leads to severe hemorrhagic conditions.^60^, ^61^ Therefore, siRNA loaded into the nano-vesicle may penetrate into this barrier and may inhibit early infection of organ-specific cytokine production that has been demonstrated promising in several reports.^62^, ^63^, ^64^

## 5 Conclusion

RNAi pathway is a significant event in biological systems in which a particular gene can be silenced or degraded through post-translational modification. This bio-molecular activity could be a possible way to silence non-structural protein encoded by the dengue virus. In the current study, we anticipated two efficient siRNA molecules against all serotypes of DENV that might be a potential and innovative therapeutics against the DENV infection. Further extensive *in vitro* studies, studies in suitable laboratory animals, and human trials are necessary to validate the efficiency of these siRNA molecules as applicable therapeutics. These findings would be a foundational piece of research for both academics and pharmaceutical implementation in future drug development.

## CRediT authorship contribution statement

**Md. Nur Islam:** Writing – review & editing, Writing – original draft, Software, Methodology, Formal analysis, Data curation, Conceptualization. **Israt Jahan Asha:** Writing – review & editing, Writing – original draft. **Aninda Kumar Gain:** Writing – review & editing, Writing – original draft. **Raihanul Islam:** Writing – review & editing, Writing – original draft. **Shipan Das Gupta:** Writing – review & editing, Writing – original draft, Conceptualization. **Md. Murad Hossain:** Writing – review & editing, Writing – original draft. **Shuvo Chandra Das:** Writing – review & editing, Writing – original draft. **Mohammed Mafizul Islam:** Writing – review & editing, Writing – original draft. **Dhirendra Nath Barman:** Writing – review & editing, Writing – original draft, Validation, Supervision, Resources, Project administration, Methodology, Investigation, Formal analysis, Data curation, Conceptualization.

## Declaration of competing interest

The authors declare that they have no known competing financial interests or personal relationships that could have appeared to influence the work reported in this paper.

## References

[1] Leung XY, Islam RM, Adhami M, et al. A systematic review of dengue outbreak prediction models: Current scenario and future directions. Poonawala H, ed. PLOS Neglected Tropical Diseases. 2023; 17(2): e0010631. Doi: 10.1371/journal.pntd.0010631. 10.1371/journal.pntd.0010631 PMC9956653 36780568

[2] Bhatt S., Gething P.W., Brady O.J. (2013). The global distribution and burden of dengue. Nature.

[3] Ponti R.D., Mutwil M. (2021). Structural landscape of the complete genomes of dengue virus serotypes and other viral hemorrhagic fevers. BMC Genomics.

[4] Stica C.J., Barrero R.A., Murray R.Z., Devine G.J., Phillips M.J., Frentiu F.D. (2022). Global evolutionary history and dynamics of dengue viruses inferred from whole genome sequences. Viruses.

[5] Gebhard L.G., Filomatori C.V., Gamarnik A.V. (2011). Functional RNA elements in the dengue virus genome. Viruses.

[6] Dwivedi V.D., Tripathi I.P., Tripathi R.C., Bharadwaj S., Mishra S.K. (2017). Genomics, proteomics and evolution of dengue virus. Brief Funct Genomics.

[7] Ligon B.L. (2005). Dengue fever and dengue hemorrhagic fever: a review of the history, transmission, treatment, and prevention. Semin Pediatr Infect Dis.

[8] Bonna A.S., Pavel S.R., Mehjabin T., Ali M. (2023). Dengue in Bangladesh. IJID One Health.

[9] Sharmin S., Viennet E., Glass K., Harley D. (2015). The emergence of dengue in Bangladesh: epidemiology, challenges and future disease risk. Trans R Soc Trop Med Hyg.

[10] Kayesh M.E.H., Khalil I., Kohara M., Tsukiyama-Kohara K. (2023). Increasing dengue burden and severe dengue risk in Bangladesh: an overview. Tropical Med Infect Dis.

[11] World Health Organization. Global strategy for Dengue prevention and control. Accessed 22 November 2024. https://iris.who.int/bitstream/handle/10665/75303/9789241504034_eng.pdf?sequence=1; 2012-2020.

[12] Ayyagari V.S. (2022). Design of siRNA molecules for silencing of membrane glycoprotein, nucleocapsid phosphoprotein, and surface glycoprotein genes of SARS-CoV2. J Genet Eng Biotechnol.

[13] Chawla P., Yadav A., Chawla V. (2014). Clinical implications and treatment of dengue. Asian Pac J Trop Med.

[14] Patel M.P., Oza V.M., Tanna H.B., Khadela A.D., Bharadia P.D., Patel J.K. (2024). Current Perspectives in Dengue Hemorrhagic Fever.

[15] Paul A.M., Shi Y., Acharya D. (2014). Delivery of antiviral small interfering RNA with gold nanoparticles inhibits dengue virus infection in vitro. J Gen Virol.

[16] Subramanya S., Kim S.S., Abraham S. (2009). Targeted delivery of small interfering RNA to human dendritic cells to suppress dengue virus infection and associated proinflammatory cytokine production. J Virol.

[17] Zhang M.M., Bahal R., Rasmussen T.P., Manautou J.E., Zhong X.B. (2021). The growth of siRNA-based therapeutics: Updated clinical studies. Biochem Pharmacol.

[18] Hall T.M.T. (2005). Structure and function of argonaute proteins. Structure.

[19] Idrees S., Ashfaq U.A. (2013). RNAi: antiviral therapy against dengue virus. Asian Pac J Trop Biomed.

[20] Rodriguez-Salazar C.A., Recalde-Reyes D.P., Bedoya J.P., Padilla-Sanabria L., Castaño-Osorio J.C., Giraldo M.I. (2022). In vitro inhibition of replication of dengue virus serotypes 1–4 by siRNAs bound to non-toxic liposomes. Viruses.

[21] Villegas-Rosales P.M., Méndez-Tenorio A., Ortega-Soto E., Barrón B.L. (2012). Bioinformatics prediction of siRNAs as potential antiviral agents against dengue viruses. Bioinformation.

[22] Tamura K., Stecher G., Kumar S. (2021). MEGA11: molecular evolutionary genetics analysis version 11. Mol Biol Evol.

[23] Notredame C., Higgins D.G., Heringa J. (2000). T-coffee: a novel method for fast and accurate multiple sequence alignment 1 1Edited by J. Thornton. J Mol Biol.

[24] Ui-Tei K., Naito Y., Takahashi F. (2004). Guidelines for the selection of highly effective siRNA sequences for mammalian and chick RNA interference. Nucleic Acids Res.

[25] Reynolds A., Leake D., Boese Q., Scaringe S., Marshall W.S., Khvorova A. (2004). Rational siRNA design for RNA interference. Nat Biotechnol.

[26] Amarzguioui M., Prydz H. (2004). An algorithm for selection of functional siRNA sequences. Biochem Biophys Res Commun.

[27] Ui-Tei K., Naito Y., Nishi K., Juni A., Saigo K. (2008). Thermodynamic stability and Watson–Crick base pairing in the seed duplex are major determinants of the efficiency of the siRNA-based off-target effect. Nucleic Acids Res.

[28] Ichihara M., Murakumo Y., Masuda A. (2007). Thermodynamic instability of siRNA duplex is a prerequisite for dependable prediction of siRNA activities. Nucleic Acids Res.

[29] Saba A.A., Adiba M., Chakraborty S., Nabi A.N. (2022). Prediction of putative potential SIRNAS for inhibiting SARS-COV-2 strains, including variants of concern and interest. Future Microbiol.

[30] Liu B., Huang H., Liao W., Pan X., Jin C., DeepSipred Y.Y. (2024). Proceedings of the 2024 4th International Conference on Bioinformatics and Intelligent Computing.

[31] Filhol O., Ciais D., Lajaunie C. (2012). DSIR: Assessing the design of highly potent SIRNA by testing a set of Cancer-Relevant target genes. PLoS One.

[32] OligoCalc K.WA. (2007). an online oligonucleotide properties calculator. Nucleic Acids Res.

[33] Reuter J.S., Mathews D.H. (2010). RNAstructure: software for RNA secondary structure prediction and analysis. BMC Bioinf.

[34] Pan W.J., Chen C.W., Chu Y.W. (2011). SIPRED: predicting siRNA efficacy using various characteristic methods. PLoS One.

[35] Rand T.A., Ginalski K., Grishin N.V., Wang X. (2004). Biochemical identification of Argonaute 2 as the sole protein required for RNA-induced silencing complex activity. Proc Natl Acad Sci.

[36] Pymol D.WL. (2002). An open-source molecular graphics tool.CCP4 Newsl. Protein Crystallogr.

[37] Pettersen E.F., Goddard T.D., Huang C.C. (2004). UCSF Chimera- a visualization system for exploratory research and analysis. J Comput Chem.

[38] Yan Y., Tao H., He J., Huang S.Y. (2020). The HDOCK server for integrated protein–protein docking. Nat Protoc.

[39] Van Der Spoel D., Lindahl E., Hess B., Groenhof G., Mark A.E., Berendsen H.J.C. (2005). GROMACS: Fast, flexible, and free. J Comput Chem.

[40] David C.C., Jacobs D.J. (2013). Principal component analysis: a method for determining the essential dynamics of proteins. Methods Mol Biol.

[41] Chan C.Y., Carmack C.S., Long D.D. (2009). A structural interpretation of the effect of GC-content on efficiency of RNA interference. BMC Bioinf.

[42] Shabalina S.A., Spiridonov A.N., Ogurtsov A.Y. (2006). Computational models with thermodynamic and composition features improve siRNA design. BMC Bioinf.

[43] Islam R., Shahriar A., Uddin M.R., Fatema N. (2024). Immunoinformatic and molecular docking approaches: siRNA prediction to silence cell surface binding protein of monkeypox virus. Beni-Suef Univ J Basic Appl Sci.

[44] Bhandare V., Ramaswamy A. (2016). Structural dynamics of human argonaute2 and its interaction with siRNAs designed to target mutant tdp43. Adv Bioinforma.

[45] Elkayam E., Kuhn C.D., Tocilj A. (2012). The structure of human argonaute-2 in complex with miR-20a. Cell.

[46] Kayesh M.E.H., Khalil I., Kohara M., Tsukiyama-Kohara K. (2023). Increasing dengue burden and severe dengue risk in Bangladesh: an overview. Tropical Med Infect Dis.

[47] Hossain M.S., Noman A.A., Mamun S.A.A., Mosabbir A.A. (2023). Twenty-two years of dengue outbreaks in Bangladesh: epidemiology, clinical spectrum, serotypes, and future disease risks. Trop Med Health.

[48] Islam M. (2024). Frequent dengue outbreaks in South Asian countries: analysis of associated risk factors and possible preventive measures. Bracuacbd.

[49] Obi J., Gutiérrez-Barbosa H., Chua J., Deredge D. (2021). Current trends and limitations in dengue antiviral research. Tropical Med Infect Dis.

[50] Liu D.Q., Lu S., Zhang L.X. (2018). An indoleamine 2, 3-dioxygenase siRNA nanoparticle-coated and Trp2-displayed recombinant yeast vaccine inhibits melanoma tumor growth in mice. J Control Release.

[51] Seyhan A.A. (2011). RNAi: a potential new class of therapeutic for human genetic disease. Hum Genet.

[52] Zhang M.M., Bahal R., Rasmussen T.P., Manautou J.E., Zhong X. (2021). The growth of siRNA-based therapeutics: Updated clinical studies. Biochem Pharmacol.

[53] Mahfuz A., Khan M.A., Sajib E.H. (2022). Designing potential siRNA molecules for silencing the gene of the nucleocapsid protein of Nipah virus: a computational investigation. Infect Genet Evol.

[54] Ojo T.O., Elegbeleye O.E., Bolaji O.Q. (2024). Hitting Epstein Barr virus where it hurts: computational methods exploration for siRNA therapy in alleviating Epstein Barr virus-induced multiple sclerosis. Neurogenetics.

[55] Reynolds C.H., Holloway M.K. (2011). Thermodynamics of ligand binding and efficiency. ACS Med Chem Lett.

[56] Urban-Klein B., Werth S., Abuharbeid S., Czubayko F., Aigner A. (2004). RNAi-mediated gene-targeting through systemic application of polyethylenimine (PEI)-complexed siRNA in vivo. Gene Ther.

[57] Ge Q., Filip L., Bai A., Nguyen T., Eisen H.N., Chen J. (2004). Inhibition of influenza virus production in virus-infected mice by RNA interference. Proc Natl Acad Sci.

[58] Buchman Y.K., Lellouche E., Zigdon S., Bechor M., Michaeli S., Lellouche J.P. (2013). Silica nanoparticles and polyethyleneimine (PEI)-mediated functionalization: a new method of PEI covalent attachment for siRNA delivery applications. Bioconjug Chem.

[59] Gupta N., Rai D.B., Jangid A.K., Pooja D., Kulhari H. (2019). Nanomaterials-based siRNA delivery: Routes of administration, hurdles and role of nanocarriers. In: Springer eBooks.

[60] Soe H.J., Khan A.M., Manikam R., Raju C.S., Vanhoutte P., Sekaran S.D. (2017). High dengue virus load differentially modulates human microvascular endothelial barrier function during early infection. J Gen Virol.

[61] Chanthick C., Kanlaya R., Kiatbumrung R., Pattanakitsakul S.N., Thongboonkerd V. (2016). Caveolae-mediated albumin transcytosis is enhanced in dengue-infected human endothelial cells: a model of vascular leakage in dengue hemorrhagic fever. Sci Rep.

[62] Eyford B.A., Singh C.S.B., Abraham T. (2021). A nanomule peptide carrier delivers SIRNA across the intact Blood-Brain barrier to attenuate ischemic stroke. Front Mol Biosci.

[63] Zhou Y., Zhu F., Liu Y. (2020). Blood-brain barrier–penetrating siRNA nanomedicine for Alzheimer’s disease therapy. Sci Adv.

[64] Zou Y., Sun X., Wang Y. (2020). Single siRNA nanocapsules for effective siRNA brain delivery and glioblastoma treatment. Adv Mater.

